# Salutatory

**Published:** 1912-07

**Authors:** 


### VITAL FACTS EVERY WOMAN SHOULD KNOW.

*Teach me the way to a strong body, a pure mind, a noble character.*

"*Ignorance is not innocence.*"

SALUTATORY.

*To the Readers of the "Red Back":*

Why have I undertaken to edit in the Texas Medical Journal a department to be devoted to the publication of *Vital Facts Every Woman Should Know?*

Let me tell you so that you will understand my motives, and, I trust, sympathize with my work.

I have for many years past numbered among my friends some of the leading physicians of the State; some broad, clear-headed ministers; some noble women, all of whose love for humanity has made them study diligently the great problems that confront us today. These men and women are agreed that much of the suffering in the world is attributable to ignorance on the part of mothers. They are unanimous in the belief that such a department is the need of the hour.

Since my advent into the home of the "Red Back" I have grieved that so few of the women should be benefited by the splendid articles that have appeared from time to time in the pages of this JOURNAL. There have been many such articles that were full of helpful information; but, alas! who read them; the doctors only, I think. It has always seemed to me a waste of good space that these articles should be written for and read by the men who know these things better than any other class of men in the world.

Hence I have decided, with the permission of the good Doctor, to use a few pages of the JOURNAL each month for articles of especial value to my sex, and to put this JOURNAL in the homes.

Now, Doctor, this does not mean that women will read *only* this department. I hope that every contributor will couch his thoughts in such simple language (whenever it is possible) that *all* the readers of the "Red Back" will understand and enjoy them. I believe, too, Doctor, that you will find new inspiration in the knowledge that, whereas heretofore your articles have been read by your brothers in the profession only, you will have lay readers who are eager and anxious to read what you have to say. To them it will not be trite.

The patent medicine venders have had a wonderfully fertile soil in which to plant their poisonous seed for what the average woman knows of the vital things of life. She has learned through the newspapers or the villainous pamphlets thrown at her door,—and thereby has been misled. I think she should have this information come from the leaders among her own sex and from clean, conscientious physicians, expressed through a high-class medical journal, rather than from patent medicine advertisements, and sentimental books on "sexology." Such books are often false and misleading, and have been written solely for the money they would bring. If you do not believe that women are reaching out for this knowledge, ask the book dealer how many books he sells on questions of sex each month.

This question of educating the woman has been vexing the minds of thinking doctors for a long time, and much good literature has been sent out in the form of circulars. But there must be system and co-operation to accomplish a work of this kind.

Doctor, the medium is here. The TEXAS MEDICAL JOURNAL has always stood for purity of thought, high ideals, progressiveness. We believe the need of the age is, that women shall know something of themselves, especially of the physiology of reproduction.

Eight years ago, when the State Medical Association did me the great honor to elect me their "Mascot," I told you then that I believed you, as a class, are the best men in the world. These years have brought no change of feeling towards you, but rather a steady conviction that as educational factors you have no equals; and is it not honor enough that in the family your word is law? To you are told secrets no minister ever hears. You are permitted to open the closet door and view the family skeleton of whose existence the good preacher is not even aware.

Now I shall succeed (or fail) just as you use your influence in the home to help me. If you believe in this work, tell your patients about it, and get them interested. Take the JOURNAL home to your wife and ask her to read it, and give it to some friend. If you wish me to send sample copies to your patients who would be interested, just make a list and send it to me, and I will send them.

If you appreciate the importance and necessity of this work, write and tell me about it. A few lines will mean much to me. Don't think some other fellow will write, and you need not do so; I want to hear from you.

The articles for this department will come from the pens of men and women who believe that the woman is the most potent factor in the shaping of human destiny.

In this issue of the JOURNAL, I am simply introducing you to my department. In the next issue will appear some of the "vital things" promised.

My staff of collaborators is not complete, but it bears the names of some of the best women and men in the United States.

JOSEPHINE DANIEL.

---

*Austin, Texas, June 2, 1912.*

*To My Friends:*

*Will you please send me the name of some nice bright woman in your town who will make a house-to-house canvass and take subscriptions for the "Red Back" on a liberal commission? I want to*

*put it into the homes. If you will do so drop me a card today while this matter is fresh in your mind.*

*MRS. F. E. DANIEL,*

*204 East Ninth St.,*

*Austin, Texas.*

*A sample copy means, "Send me your Subscription."*

---

SOME EXPRESSIONS OF APPROVAL.

TEXAS STATE BOARD OF HEALTH.

AUSTIN, TEXAS, June 24, 1912.

*Mrs. F. E. Daniel, Austin, Texas.*

DEAR MRS. DANIEL: It is with a great deal of gratification that I note your intention of conducting a department in the "Red Back" for instruction along the lines of sex and personal hygiene. This is in line with the work undertaken by our State Health Bulletin.

The time has come when false modesty attempts to keep from growing children a helpful knowledge of sex hygiene and should be put aside. The danger of permitting them to acquire, unguided, the knowledge that comes with physical development must be overcome by the intelligent work of the mother.

This work you are undertaking meets with my hearty endorsement, and I wish you every success in the undertaking.

Very truly yours,
RALPH STEINER, M. D.,
State Health Officer.

---

Mrs. J. A. Jackson, President Texas Woman's Press Association, said: "Ignorance is sin; sin is death."

---

Dr. Grace writes:

"SEGUIN, TEXAS, June 5, 1912.

"Regarding the use of a few pages of the 'Red Back' for the enlightenment of women, I believe it will do great good, if it can reach the women. A campaign of education along these 'tabooed' lines is more needed than anything else I know, for the public welfare and the moral life of our people. Let the sunlight of knowledge in on the sex questions,—dispel the mystery, and many mile-posts toward morality will have been passed. If you can be instrumental in lifting the veil of ignorance from these important subjects,—from the eyes of your sisters, your name will be blessed.

"M. B. GRACE, M. D."

"Your idea is an inspiration. You will find that the women are eager for knowledge.

"MARGARET HOLLIDAY, M. D."

